# A New Method for Total Fat Detection in Raw Milk Based on Dual Low-Coherence Interferometer

**DOI:** 10.3390/s19204562

**Published:** 2019-10-20

**Authors:** Abraham Gastélum-Barrios, Genaro M. Soto-Zarazúa, Juan F. García-Trejo, Juan M. Sierra-Hernandez, Daniel Jauregui-Vazquez

**Affiliations:** 1 Facultad de Ingeniería Campus Amazcala, Universidad Autónoma de Querétaro, Carr. Chichimequillas S/N Km 1, Amazcala, El Marqués, Querétaro 76265, Mexico; abraham.gastelum@uaq.mx (A.G.-B.); fernando.garcia@uaq.mx (J.F.G.-T.); 2 Departamento de Electrónica, División de Ingenierías, Campus Irapuato-Salamanca, Universidad de Guanajuato, Carretera Salamanca-Valle de Santiago Km. 3.5+1.8 Comunidad de Palo Blanco, Salamanca, Guanajuato 36787, Mexico; jm.sierrahernandez@ugto.mx

**Keywords:** optical fat sensor, Fabry–Perot interferometer, raw milk, phase modulation

## Abstract

The present work experimentally demonstrates a multimode fiber optic sensing setup for total fat detection in raw milk samples. The optical fiber arrangement incorporates a low-coherence Fabry–Perot cavity operating in dual response. The system provides a phase modulation for a total fat range from 0.97 to 4.36%. Here, the protein remains constant at ≈3%. The data indicate that maximum sensitivity close to 616 pm/%fat could be achieved at optimal wavelength operation (500 nm). In addition, the system presented a minimal repeatability error measurement of 0.08%, cross-sensitivity between protein and fat of 0.134, and a regression coefficient of $r^{2}=0.9763$. A thermal analysis was also performed, which indicate the temperature immunity of the system. The proposed method represents a low-cost alternative to detect minimal fat variations in raw cow milk.

## 1. Introduction

In the dairy industry, total fat and protein content has an important role in determining the quality and price of milk [1]. During the past few decades, several techniques have been widely studied to determine these milk quality indicators, and two main branches are found in the literature: chemical analysis and optical instruments. The former requires specialized laboratories that involve costly equipment and materials, as well as highly trained staff. Therefore, development has been focused on techniques that do not require chemical agents. The second is based on the interaction between light and milk, and many proposals have been reported [2,3,4,5,6,7]. One of the most attractive techniques is based on spectroscopy [8]. This technique analyzes the transmission spectra of the light that crosses the liquid sample. By using the Beer–Lambert–Bouguer law, it is possible to obtain the absorbance and correlate compounds from the sample [9]. Despite this method being widely used, the technique has undesired scattering effects produced by optically dense liquids like milk [9]. On the other hand, spectroscopy techniques can be combined with optical fiber methods using different wavelength operations such as ultraviolet (UV), visible (VIS), or near-infrared (NIR) spectra. As a result, different milk properties are exploited. The use of optical fiber sensors has been increased since several reported techniques have assured that sensitivity can be improved [10]. Some of the reported works use an NIR source and the transmission spectra is analyzed [11,12], while other fiber optic works use the relationship between the refractive index (RI) and total fat content [10]. It is important to recall that the size of fat globules (average diameter of 3 µm) and protein particles (average diameter of 120 nm) produce scattering effects [11]. When a milk sample is homogenized, the size of the fat globules is around 1 µm [13]. Moreover, the RI decreases as the fat content diminishes [10]. In addition, according to our literature review related to fat milk estimation, most works are based on intensity modulation to determine the total fat in raw cow milk. Here, the demodulation process is sensitive to fiber bending and power source variations. In contrast, phase modulation offers immunity to the undesired effects presented by intensity modulation. However, to the best of our knowledge, only a few works have employed this modulation to detect total fat in raw cow milk. One technique that allows detection of chemical changes by phase modulation is based on all-fiber-optic interferometers, such as Fabry–Perot [14], Mach–Zehnder [15], Michelson [16], and Sagnac [17] interferometers. Among them, the extrinsic Fabry–Perot interferometer (EFPI) is a popular configuration due to its advantages, which include non-complex implementation, cost-effectiveness, and reflection mode operation. However, some disadvantages are calibration operation, a complicated demodulation process, and temperature sensitivity.

This work presents a multimode optical fiber sensing setup based on dual response EFPI to detect total fat content in milk. The scheme provides an interference spectrum, and the milk samples generate a wavelength shifting at a specific region which makes it possible to detect fat fluctuations. The system underwent temperature and repeatability tests, presenting immunity to both external and internal temperature changes in conjunction with minimal error estimations.

## 2. Materials and Methods

### 2.1. Experimental Setup

**Figure 1:**
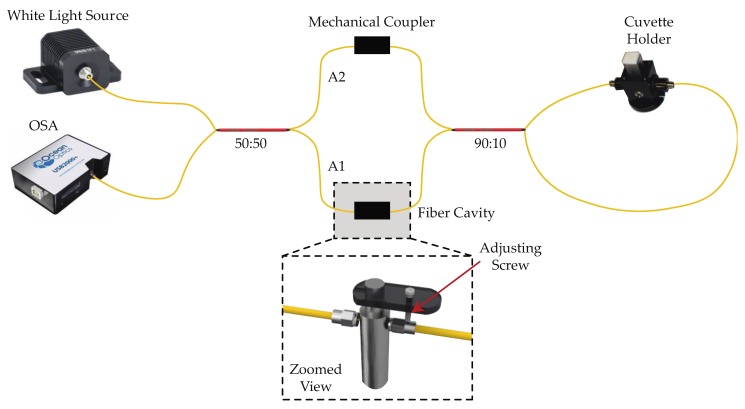
Schematic diagram of the experimental setup. OSA: optical spectrum analyzer.

The experimental setup is shown in Figure 1. Here, a fiber-coupled LED (MBB1F1, Thorlabs Inc., USA) provided a visible broadband signal from 470 to 850 nm with a T-Cube LED driver (LEDD1B, Thorlabs Inc., Newton, NJ, USA) in continuous mode. This signal was launched into the system using a 3-dB 2 × 2 multimode fiber coupler (TM200R5S2A, Thorlabs Inc., Newton, NJ, USA). The signal from the source went to the coupler and split into two arms (A1 and A2). The A1 arm had an extrinsic low-coherence Fabry–Perot cavity formed by two SMA905 (Thorlabs Inc., Newton, NJ, USA) optical fiber connectors.

The A2 arm had an inline mechanical optical fiber connector. The transmitted light from the EFPI and the A2 were coupled into an unbalanced loop fiber, where the loop was implemented using a 90:10 2 × 2 optical fiber coupler (TM105R2S2A, Thorlabs Inc., Newton, NJ, USA). At this point, a cuvette holder (CUV-FL-DA, Ocean Optics Inc., Largo, FL, USA) was set in the middle of the loop. The cuvette holder was composed of two GRIN lenses of 5 mm diameter and 10 mm focal length located on both sides of the holder. The white light was focused directly on the milk sample, and the transmitted spectrum was focused onto the optical fiber. The recirculating light from the loop returned to arms A1 and A2and was then combined by an initial fiber coupler. The system response was monitored by the residual port of the 3-dB coupler, which in this case was an optical spectrum analyzer (OSA) (USB2000+, Ocean Optics Inc., Largo, FL, USA). The data provided by the OSA was processed by a computer.

### 2.2. Sample Preparation

Sixteen experimental samples were obtained from manually milked cows, and were refrigerated at 4 °C for one day. The samples were warmed to 37 °C (the temperature of milk when it leaves the udder [18]) and homogenized for 20 min using an ultrasonic bath (Bransonic 2800 MH, Branson Ultrasonic, Danbury, CT, USA). Then, using a commercial milk analyzer (Lactoscan S, Milkotronic Ltd., Nova Zagora, Bulgaria) total fat and protein content was determined. The samples were tested using the described schema in order to detect fat content changes. Their compositions are shown in Table 1; note that most samples had nearly constant protein content.

### 2.3. Theoretical Model

**Figure 2:**
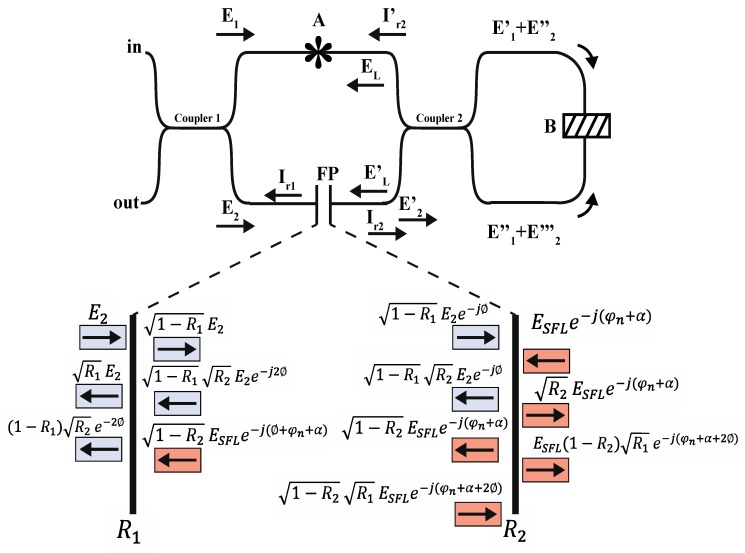
Fiber optic arrangement sketch and its optical path analysis. The mechanical coupler is represented by **A**, and **B** represents the cuvette holder with the milk samples. Inset: Fabry–Perot dual response analysis.

The proposed method is based on a dual low-coherence Fabry–Perot cavity [14,19,20]. The optical paths and light interaction are presented in Figure 2. The light from the source is split into two beams ($E_{1}$ and $E_{2}$) by the 3-dB optical fiber coupler (coupler 1). Hence, $E_{1}$ goes to the second coupler (by A in Figure 2) without change. Meanwhile, $E_{2}$ passes through a one-layer EFPI, where one part of the light is transmitted ($E_{2}^{\prime }$), and the rest is reflected. According to inset in Figure 2, when the electric field $E_{2}$ interacts in the Fabry–Perot cavity, it generates a low-coherence interference spectrum ($I_{r1}={\left|E_{2r}/E_{2}\right|}^{2}$) described by:

(1) $$I_{r1}=R_{1}+{\left(1-R_{1}\right)}^{2}R_{2}+2\left(1-R_{1}\right)\sqrt{R_{1}R_{2}}\left(cos2\phi \right).$$

The interference is generated by two aligned SMA905 optical fiber connectors. In our case, the separation between both connectors was close to 8 µm. The reflectivity ($R_{1}$ and $R_{2}$) is generated by the refractive index interfaces, where the RI of the optical fiber $n_{F}$ (1.458) and the RI of the material in the cavity $n_{c}$ (air = 1.0) provide the reflections governed by:

(2) $$R=R_{1}=R_{2}={\left(\frac{n_{F}-n_{c}}{n_{F}+n_{c}}\right)}^{2}.$$

We considered both reflections to be similar because the same refractive indices were involved in the cavity interfaces. The phase of the interference generated by the Fabry–Perot cavity can be expressed by:

(3) $$\phi =\frac{2\pi n_{c}L}{\lambda },$$

where $n_{c}$ represents the refractive index of the cavity, L is the length of the cavity, and the wavelength of the incident light is λ. In our system, once the low-coherence spectrum is generated, the transmitted $E_{1}$ and $E_{2}^{\prime }$ are coupled by the 90:10 optical fiber coupler (coupler 2). As a result, two intensity electric fields are generated by the corresponding sum of the input signals: $E_{L}=E_{1}^{\prime }$ + $E_{2}^{\prime \prime }$ and $E_{L}^{\prime }=E_{1}^{\prime \prime }$ + $E_{2}^{\prime \prime \prime }$.

**Figure 3:**
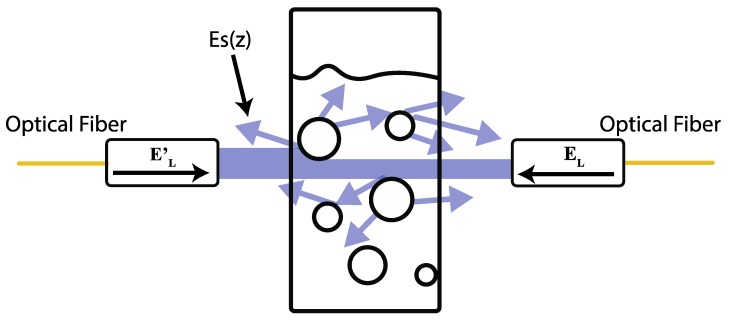
Visual representation of the incident and scattered beams through the milk sample into the quartz cell.

These electric fields are recirculated in opposite directions into the fiber loop, composed by closing the 90:10 fiber coupler using the cuvette holder shown in Figure 1. At this point, the light reaches B where the light–milk interaction generates scattering, refractive index, and absorbance effects [9,21,22,23]. Because the sample is illuminated in two directions, multiple scattering rays are exited and the absorbance is presented in all scatter directions (see Figure 3) before the extinction is presented. Note that the refractive index and scattering are attributed to the fat globules and protein micelles [9]. The size of the fat globules after homogenization is greater than the light wavelength used in the present study, which makes Mie’s scattering theory applicable [24]. As a result, multiple scattered beams are generated, and the signal result is focused back to the optical fiber system. The scattering electric field can be described by [25]:

(4) $$E_{s}\left(z\right)=E_{O}\frac{k^{2}}{z}\left(\overset{↔}{l}-\hat{k}\hat{k}\right)\overset{N}{\underset{n=1}{\sum }}e^{j\varphi_{n}}\alpha_{n}\cdot E_{n},$$

where $\overset{↔}{l}$ and $\hat{k}\hat{k}$ are the perpendicular direction and the resulting product of the scattered light, respectively. The scattered electric field generated and the polarizability tensor are represented by $E_{n}$ and $\alpha_{n}$. The distance vector from the scattered electric field to the detector and the incident wavelength are considered by z and λ, respectively. The phase between the beams generated is defined by $\varphi_{n}=\left(\mu_{s1}\chi_{s1}+\mu_{s2}\chi_{s2}\right)d$ [26], where d is the cuvette length, $\mu_{s1}$ is the scatter fat coefficient, and $\chi_{s1}$ represents the fat concentration, respectively. Meanwhile, $\mu_{s2}$ and $\chi_{s2}$ depict the protein information (scatter coefficient and concentration, respectively). Owing to the simultaneous incident of $E_{L}$ and $E_{L}^{\prime }$, several forward and backward scatter electric fields ($E_{s}\left(z\right)$) are generated. These beams, governed by Equation (5), are focused back into the fiber optic loop (see Figure 3). It is important to recall that absorption and scattering are strongly related to the size of the fat globules and protein micelles.

The scatter electric field into the loop excites several fiber optic modes. These modes recirculate into the loop. The electric field of the modal trajectories into the loop can be described by [27]:

(5) $$E_{SFL}\left(z\right)=E_{O}\frac{k^{2}}{z}\left(\overset{↔}{l}-\hat{k}\hat{k}\right)\overset{N}{\underset{n=1}{\sum }}\overset{M}{\underset{m=1}{\sum }}\alpha_{n}\cdot E_{n}\cdot C_{nm}e^{j(\varphi_{n}+\alpha )},$$

where $C_{nm}$ is the coupling coefficient of the modes involved and α is the phase between the modal trajectories into the unbalanced fiber loop. The observation of the interference effect is limited by several factors, such as the large number of modes, short loop length (1.6 m), unbalanced coupling ratio (90:10), and wavelength operation (400–800 nm) [27]. However, when the electric field $E_{SFL}\left(z\right)\;$ arrives to the EFPI by the residual port of coupler 2, one part of the light is transmitted and the rest is reflected to the loop. An interference spectrum is generated as a result: $I_{r2}={\left|E_{SFLr}/E_{SFL}\right|}^{2},$

(6) $$I_{r2}=R_{1}+{\left(1-R_{1}\right)}^{2}R_{2}+2\left(1-R_{1}\right)\sqrt{R_{1}R_{2}}\left(\cos\left((2\phi +\varphi_{n}+\alpha \right)\right).$$

It is important to mention that the intensity variations of the electric vector are strongly related to the reflectivities in the EFPI cavity. The interferences generated $(I_{r1}$ and $I_{r2})$ are provided by the EFPI and the same cavity generates a dual response by the electric field directions [20]. However, when the interference signal is circulating into the loop, the light is once again affected by the milk solution. Nonetheless, the trajectories described in Figure 2 are the only ones considered for the phase contribution. Because $I_{r2}$ is directed to coupler 1 by fiber arrangement, the total reflected signal captured by the OSA can be expressed by the relation: $It=I_{r1}+I_{r2}$,

(7) $$It=2\left[R+{\left(1-R\right)}^{2}R\right]+2\left(1-R\right)R\left(\cos\left(2\phi \right)+\cos(2\phi +\varphi_{n}+\alpha )\right).$$

**Figure 4:**
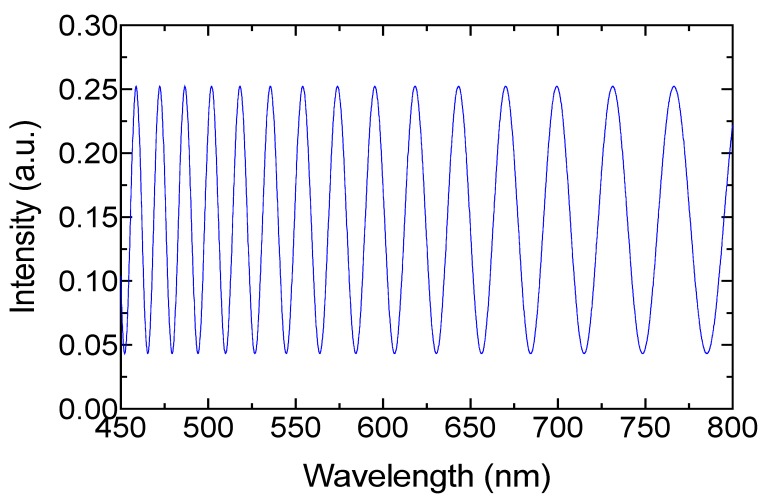
Output spectrum of the approximated model.

To simplify the expression, both reflections were considered to have the same value. The interference generated is related to the phase contribution by the milk scattering and the fiber loop [26]. Despite the fiber loop providing a minimal contribution, we considered this phase because it is the environment where the scatter light is circulated. Because of the RI of the optical fiber and air mentioned before, and because the length of the cavity was 8 µm, this was performed using an approximation of the mathematical model described in Equation (1). In Figure 4, the output signal where the free spectral range Δλ (FSR) was from 13.2 to 36.8 nm can be observed. The approximation was computed without considering $\varphi_{n}$ and α, and only the dual response of the EFPI was integrated into the model simulation. It is important to recall that the polarization effects are presented by the light–milk interaction and these can be studied by the scattering matrix [22], where the intensity is only considered in the Stokes’ vectors. However, in this work, we were focused on employing the phase modulation to detect total fat in raw cow milk.

## 3. Results and Discussion

**Figure 5:**
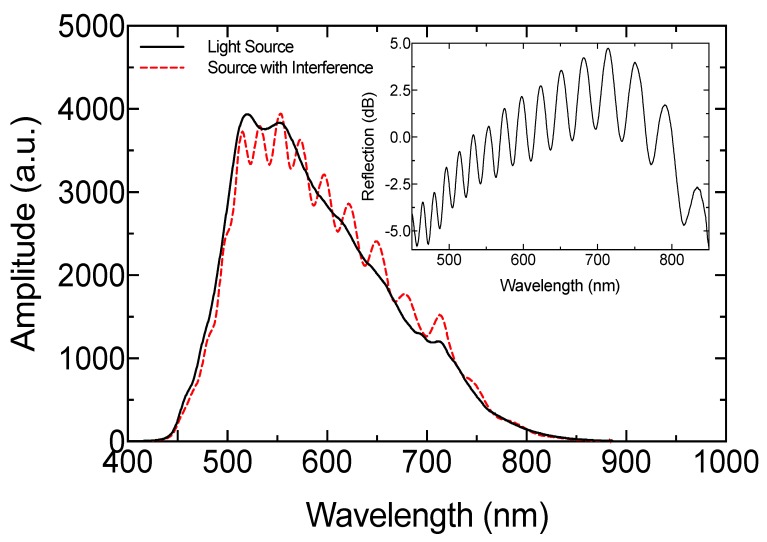
Reflected spectrum of the light source and optical interference response. Inset is the reflection signal.

The response of the sensing setup, without cuvette and milk samples, is presented in Figure 5. The interference spectrum had maximum and minimum visibility around 5.03 and 1.55 dB, respectively. Moreover, the reflected signal in some parts had an intensity increment. This is because of the metal optical fiber connector surface. The reflection spectrum exhibited an oscillating FSR, where the minimum and maximum values were 17.77 and 35.13 nm—very close to the previously described approximation model.

**Figure 6:**
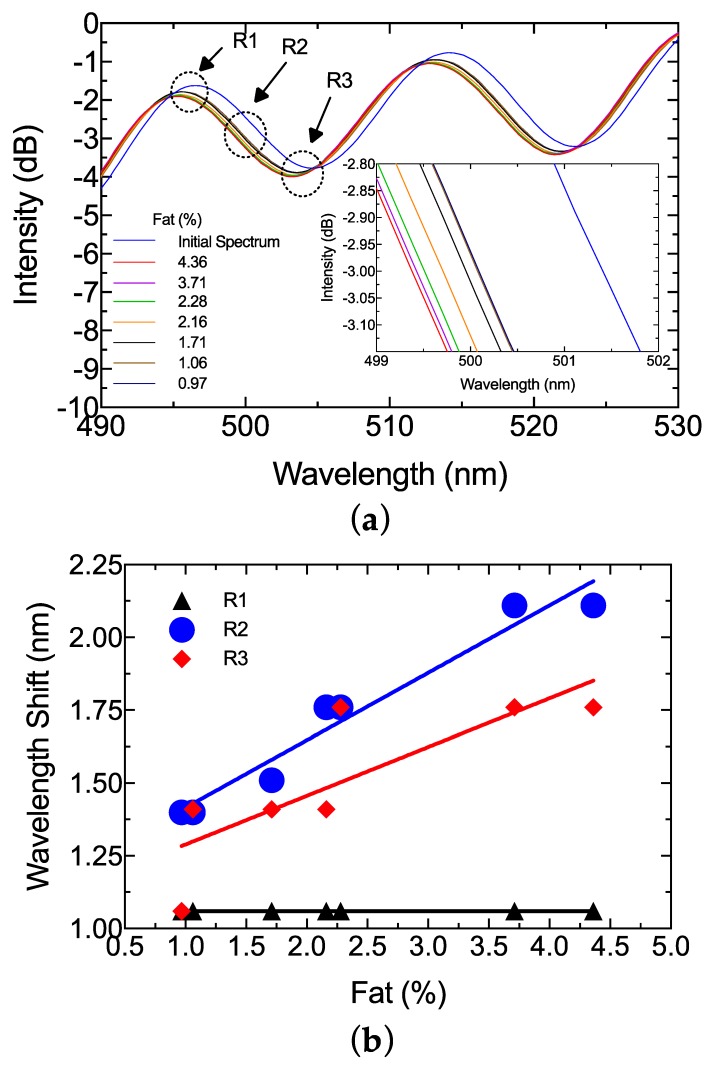
(**a**) Interference spectrum response for total fat variation. Inset is a zoomed view of the middle region (R2). (**b**) Wavelength shift analysis of the three regions with linear regression.

**Figure 7:**
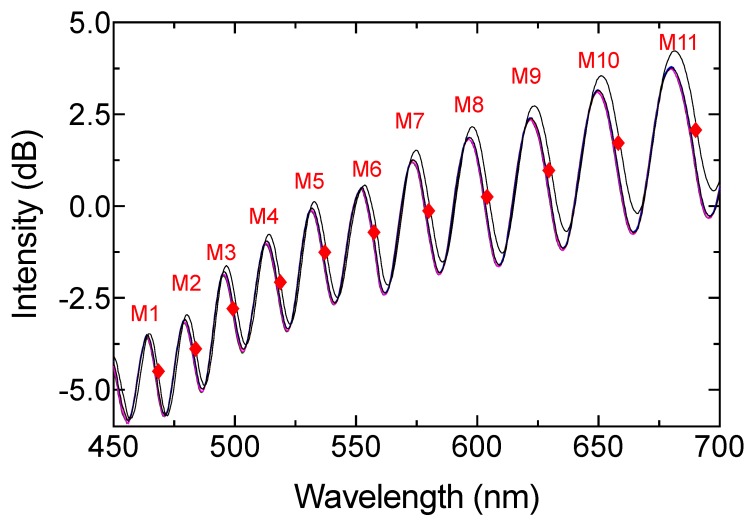
Middle regions of the visible interferogram.

The milk samples presented in Table 1 were introduced into the system using a quartz cuvette with 1 mm path. The reflection response is presented in Figure 6. A blueshift of the spectrum was observed as fat increased (see inset Figure 6a). According to Figure 6b, the signal had a different response according to the peak (R1), deep (R3), and middle points (R2). The R1 region presented a linear response without a wavelength shifting between the different milk samples. The wavelength only shifted between samples and the initial spectrum. The R3 region had nonlinear wavelength variation, and only unstable power variation was observed. However, the middle point (R2) between peak and deep presented a clear wavelength shift. A linear response could be achieved in this region (see Figure 6b). According to the previous section, the phase of the reflection spectrum depends on the Fabry–Perot cavity and the OPD. In this work, scatter and refractive index changes were related to the wavelength operation [4,21,22,23,27,28]. As a result, all the R2 regions were analyzed to provide an optimal wavelength operation (see Figure 7). A second-order regression was used to study the best wavelength operation, and the sensitivity was also analyzed for each region. The data obtained from the study are presented in Table 2. According to the proposed approximation, the best region to detect total fat in raw cow milk is the M3 zone, which operated at approximately 500 nm. This shifting response presented a square adjustment of 0.9739 and sensitivity close to 0.616 nm/%fat.

Despite the regression adjustment from region M2 being equal to 1, this wavelength range was not considered for signal analysis due to the absence of wavelength shift and because all the milk samples were superimposed on each other. The optimal wavelength operation has been predicted by prior work [3,5,7,29]. However, to the best of our knowledge, most of these are based on the intensity modulation and transmitted spectrum, which present typical cons. According to our results, the system could be reduced by incorporating a cyan color fiber-coupled LED and photodetector, where responsivity plays an important role in optimizing the sensing setup.

**Figure 8:**
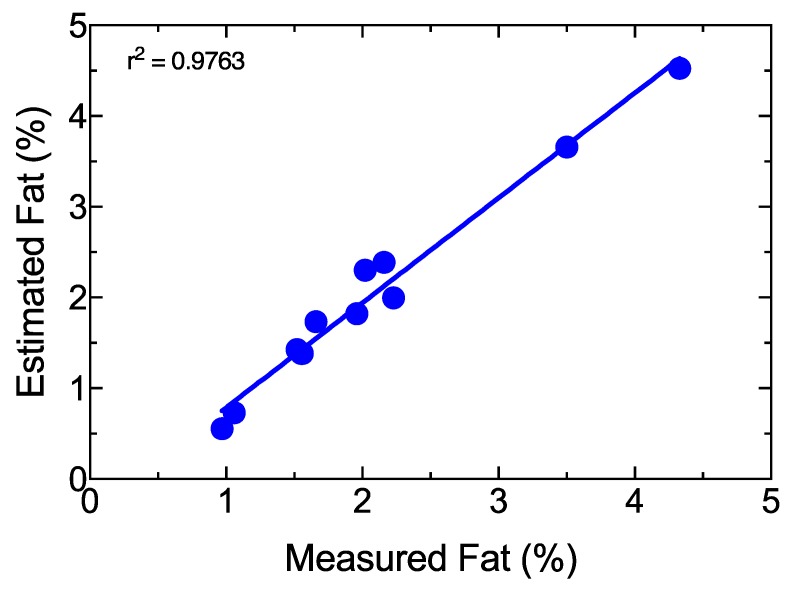
Validation results of the fat model.

**Figure 9:**
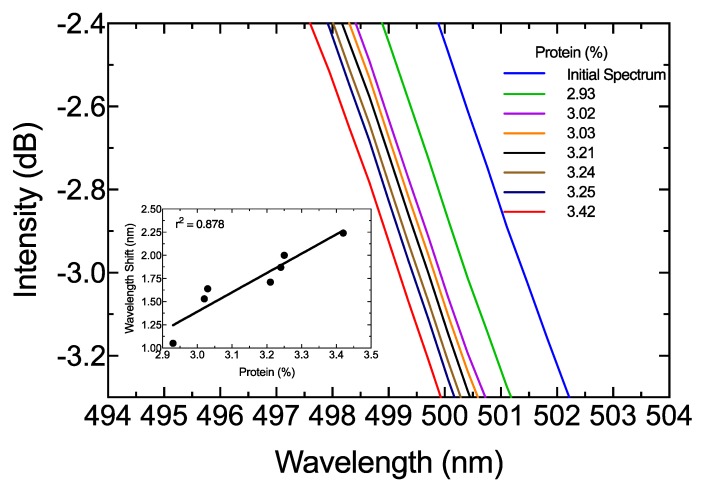
Zoomed-in view of the R2region for protein estimation. Inset is the square adjustment of the wavelength shift from the initial spectrum as reference.

In order to test the capability of estimating fat content in raw milk, 11 different samples were analyzed with the proposed schema, and wavelength shift in the 500-nm M3 region between milk samples and initial spectrum was measured. Figure 8 shows the experimental results, with a square adjustment of 0.9763. A one-way ANOVA was also performed with a confidence level of 95% and a *p*-value of 0.8735. This means that predictions were not significantly different from real measurements. Because the purpose of this study was to validate the sensing system for total fat detection, the protein variation response was also analyzed. A set of seven samples with similar fat content (around 2%) were selected. A similar analysis to the one described above for fat determination was performed. The results are shown in Figure 9. The square adjustment from the selected samples was 0.878 and sensitivity was close to 4.571 nm/%protein. Therefore, the cross-sensitivity between protein and fat concentration was equal to 0.134.

**Figure 10:**
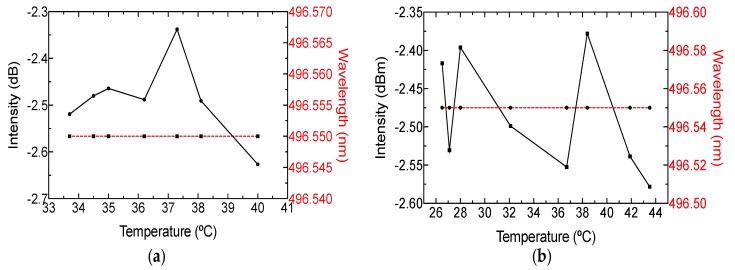
Power and wavelength response for optimal peak wavelength operation when (**a**) milk temperature and (**b**) external temperature were modified.

It is important to stress that milk has a temperature close to 37 °C when it is milked from the cow, but this temperature decreases over time. Therefore, it is important to study the thermal effect in the sensing setup. The temperature of the milk samples was estimated using an infrared thermometer (FLUKE-59 MAX, Fluke Corp., Everett, WA, USA). The wavelength and power disparities are presented in Figure 10a. Noted that wavelength was not altered as the temperature changed, but the intensity suffered an instability close to 0.2 dB. Because the intention was to test external temperature changes, the optical arrangement was analyzed by varying the temperature near the cuvette holder using a hot plate. It can be seen in Figure 10b that external temperatures between 26 and 44 °C did not affect the phase spectrum. Nevertheless, the intensity was altered around 0.1 dB. It is important to mention that power instabilities were related to the scattering effect. This behavior can assure that the system is stable to milk temperature.

**Figure 11:**
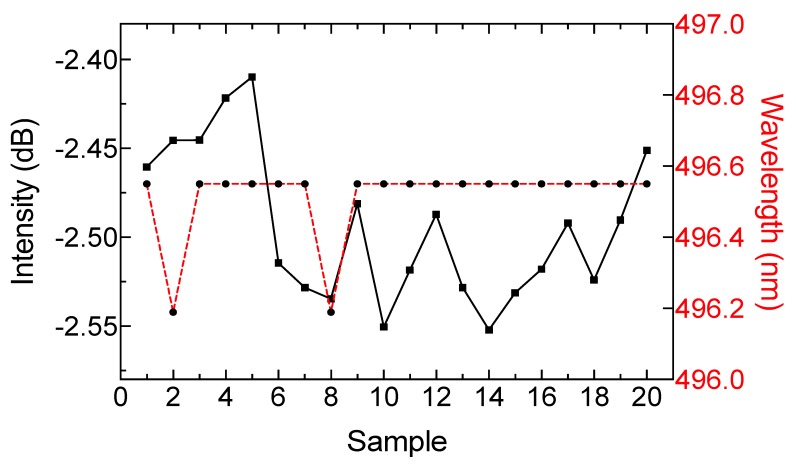
Comparison of intensity and wavelength displacement in the repeatability test.

To test repeatability, a milk sample with a fat content of 4.36% was taken at constant temperature and measured with the optical system 20 times. After each measurement, the optical system remained without a sample for five minutes. In Figure 11, the intensity and wavelength variations are presented for each repetition. The results indicate that power variation around 0.15 dB can be expected and the wavelength shift presented a relative error close to 0.08%.

Several studies have been focused on the analysis of raw milk based on different approaches, wavelength ranges, and configurations. The present study was based on the VIS range, in addition to the reflectance phenomena and phase modulation. To the best of our knowledge, there is no study based on this approach. However, this technique can be compared to studies based on optical properties of light. Table 3 shows related and outstanding studies.

The study closest to this proposal is the one by [11], where the authors present a reliable method to implement a laboratory analysis based on the reflectance spectrum at 1060 nm. However, a small range of fat (0.17–0.22%), and the error in the determination of 3%, were presented. It is necessary to note that all the studies reviewed were based on intensity modulation of the spectrum, either for transmission, reflectance, absorbance, or scattering phenomena. Nevertheless, these kinds of analyses are sensitive to fiber bending and power-source variations.

## 4. Conclusions

A new method to detect small variations of fat in raw milk was experimentally validated. The method employs a multimode fiber optic arrangement with a one-layer extrinsic Fabry–Perot interferometer, which generated a dual response when different light directions were induced. The system generated an optical fiber interference with an increased FRS from 17.77 to 35.13 nm, and maximum and minimum visibility of 5.03 and 1.55 dB, respectively. The interference spectra were analyzed by increasing the total fat percentage in the milk sample. The total fat varied from 0.97% to 4.36%. As a result, an optimal wavelength operation close to 500 nm was determined. The FPI provided a blueshift of the spectrum as the total fat sample increased. Here, a maximum sensitivity of 0.616 nm/%Fat was achieved. The experimental results showed that the proposed method could estimate fat content in raw milk samples with an $r^{2}=0.9763$ by a second-order regression. Protein analysis was also performed, and found cross-sensitivity to the fat concentration of 0.134. Furthermore, the repeatability studies indicated a minimal error measurement close to 0.08%. In addition, the thermal analysis indicated that the system presents immunity to temperature variations, in both the sample and the system. To the best of our knowledge, the use of phase modulation to detect milk properties is limited, and the present manuscript represents a reliable alternative to detect small fat variations using phase modulation. Further studies are needed to implement this method in a dedicated system, adding signal processing techniques and calibration models.

## Figures and Tables

**Table 1:** Milk samples used in the experiment.

Sample	Fat (%)	Protein (%)
F1	0.97	3.16
F2	1.06	3.17
F3	1.71	3.05
F4	2.16	3.21
F5	2.28	3.20
F6	3.71	3.08
F7	4.36	3.23

**Table 2:** Second-order regression adjustment of the middle region through visible interferogram.

Region	R Square
M1	0.9558
M2	*
M3	0.9739
M4	0.8118
M5	0.9157
M6	0.8118
M7	0.8484
M8	0.7077
M9	0.8935
M10	0.9135
M11	0.9158

* No wavelength shifting presented.

**Table 3:** Comparison of similar research in literature.

Milk Constituent	Acquisition Method	Spectral Range (nm)	Sensitivity	Validation		Reference
				R^2^	RMSEP *	
Fat	Light Scatter Digital Imaging	400–700	-	0.973	-	[5]
Protein			-	0.974	-	
Fat	Absorbance Refractive Index	400–800	0.15 Abs/%fat	-	-	[10]
Fat	Light Scatter	1300–1400	-	0.975	-	[4]
Fat	Transmission	2500–25,000	-	0.91	0.045	[2]
Protein			-	0.801	0.02	
Fat	Reflectance	200–2000		0.984	0.00265	[11]

* RMSEP: Root-mean-square error of prediction, expressed in percentage.
